# Enhanced Efficacy of Peroxyacetic Acid Against *Listeria monocytogenes* on Fresh Apples at Elevated Temperature

**DOI:** 10.3389/fmicb.2019.01196

**Published:** 2019-06-18

**Authors:** Xiaoye Shen, Lina Sheng, Hui Gao, Ines Hanrahan, Trevor V. Suslow, Mei-Jun Zhu

**Affiliations:** ^1^ School of Food Science, Washington State University, Pullman, WA, United States; ^2^ Department of Food Science, Zhengzhou University of Light Industry, Henan, China; ^3^ Washington Tree Fruit Research Commission, Wenatchee, WA, United States; ^4^ Department of Plant Sciences, University of California, Davis, Davis, CA, United States

**Keywords:** apples, *Listeria monocytogenes*, peroxyacetic acid, antimicrobial, temperature

## Abstract

Peroxyacetic acid (PAA) is the most commonly used antimicrobial in spray bar antimicrobial treatment during fresh apple packing and processing. However, there are limited data regarding its practical efficacy against *Listeria monocytogenes* on fresh apples. This study evaluated the antimicrobial activity of PAA against *L. monocytogenes* on fresh apples applicable to current industry practice, and further examined practical parameters impacting its efficacy to maximize the biocidal effects. Apples were inoculated with a three-strain *L. monocytogenes* cocktail at ~6.0 Log_10_ CFU/apple and then subjected to comparative antimicrobial treatments after 48 h post-inoculation. An 80 ppm PAA treatment, at 30-s and 2-min exposure, reduced *L. monocytogenes* on fresh apples by ~1.3 or 1.7 Log_10_ CFU/apple, respectively. The anti-*Listeria* efficacy of PAA was not affected by the water hardness and pH of PAA solution, while it improved dramatically when applied at elevated temperature. A 2-min exposure of 80 ppm PAA at 43 and 46°C resulted in a 2.3 and 2.6 Log_10_ CFU/apple reduction, respectively. A 30-s contact time of 80 ppm PAA at 43–46°C reduced *L. monocytogenes* on apples by 2.2–2.4 Log_10_ CFU/apple. Similarly, PAA intervention at elevated temperatures significantly strengthened its effectiveness against naturally occurring apple microbiota. PAA treatment at 43–46°C can provide a vital method to improve antimicrobial efficacy against both *L. monocytogenes* and indigenous microbiota on fresh apples. Our data provide valuable information and reference points for the apple industry to further validate or verify process controls.

## Introduction

Apples are the second most commonly consumed fruit in the United States (US) and produced on more than 325,000 acres, yielding 33 billion apples annually (USAA, 2018). The average apple consumption in the United States is 22 kg per person annually and the major commercial varieties are Granny Smith, Fuji, Gala, and Red Delicious (USAA, 2017). The recent outbreak of *Listeria monocytogenes* linked to caramel apples (Angelo et al., 2017) and multiple *L. monocytogenes* recalls associated with apple products (FDA, 2015, 2016, 2017b) have brought critical concerns to the apple industry and the general public regarding control of this pathogen on fresh apple fruit surfaces during production, storage, and packing. *L. monocytogenes* is an important foodborne pathogen that causes ~1,600 hospitalization and ~260 deaths in the US annually (FDA, 2012). It can survive on fresh apple surfaces for an extended period during cold storage (Sheng et al., 2017). If contaminated apples are used in the confectionary industry, *L. monocytogenes* proliferates in the microenvironment created between the apple surface and caramel coating layer (Glass et al., 2015).

During postharvest processing and handling, antimicrobial interventions have long been employed to reduce foodborne pathogens on apples and prevent or minimize cross-contamination during wash-processing. Chlorine is the most widely used antimicrobial in the fresh produce industry, but has limited efficacy against *L. monocytogenes* at the commonly used dose range of 50–200 ppm at 1–2 min exposure (Abadias et al., 2008). Chlorine wash at 100 ppm for ~1 min only provided ~1 log reduction of *L. monocytogenes* on apples (Beuchat et al., 1998; Rodgers et al., 2004). In addition, chlorine reacts with organic matter to form carcinogenic trihalomethanes (Brown et al., 2011) and chlorates (Gil et al., 2016), giving rise to health concerns. Therefore, the fresh produce industry is actively looking for alternative chemicals and/or intervention methods with a higher antimicrobial efficacy and a lower reactivity with organic matter.

Peroxyacetic acid (PAA) is generated as an equilibrium mixture between acetic acid and hydrogen peroxide in aqueous solution, and has a strong oxidation potential of 1.81 electronic volts (Dell’Erba et al., 2007; Carrasco and Urrestarazu, 2010; Hua et al., 2011). It is approved to be used at 80 ppm as a wash water processing aid on fresh produce without further rinse requirement (FDA, 2017a). PAA has a relatively low reactivity with organic matter, compared with chlorine (Banach et al., 2015), and the formed by-products have little or no toxicity (Monarca et al., 2002a,b). In addition, PAA decomposes to harmless acetic acid and oxygen (Gehr et al., 2003). PAA has been used in the fresh produce industry to control microbial contamination in iceberg lettuce, mung bean sprouts, cantaloupe, and others (Gonzalez et al., 2004; Hellstrom et al., 2006; Wang et al., 2006; Neo et al., 2013). PAA is currently the most commonly used antimicrobial in spray bar rinse-treatment during fresh apple packing and processing according to our survey of apple packers in Washington. In spite of its popularity, however, the sparse information available indicates that PAA has a limited efficacy against *L. monocytogenes* on fresh apples. PAA at 80 ppm, applied for 80-s contact time, resulted in ~1.0 Log reduction of *L. monocytogenes* on Golden Delicious apples (Rodgers et al., 2004). A 1-min treatment of apple plugs with 80 ppm PAA resulted in ~0.8 Log_10_ CFU/plug reduction of *L. monocytogenes* (Abadias et al., 2011).

The objectives of this study were to evaluate the antimicrobial efficacy of PAA against *L. monocytogenes* and resident microorganisms on fresh apples, and further optimize parameters that are applicable to the fresh apple industry to maximize its antimicrobial efficacy.

## Materials and Methods

### Bacteria Strains

*L. monocytogenes* strains [NRRL B-57618 (1/2a), NRRL-33466 (1/2b) and NRRL B-33053 (4b)] were obtained from USDA-ARS culture collection [National Center for Agricultural Utilization Research (NRRL), Peoria, IL, US]. All strains were maintained at −80°C in Trypticase Soy Broth [Becton, Dickinson and Company (BD), Sparks, MD, US] supplemented with 0.6% yeast extract (Fisher Scientific, Fair Lawn, NJ, US; TSBYE) and 20% (v/v) glycerol.

### Preparation of Inoculum

Each *L. monocytogenes* strain was twice activated in TSBYE at 37°C for 24 h individually, then centrifuged at 8,000 × *g* for 5 min at 4°C. The resulting bacterial pellets were washed once and then resuspended in phosphate-buffered saline (PBS, pH 7.4) to achieve the target population. To prepare a 3-strain *L. monocytogenes* inoculum cocktail, each strain suspension at ~5 × 10^8^ CFU/ml was mixed at 1:1:1 ratio to ~ 6.0 Log_10_ CFU/ml in PBS for apple inoculation.

### Apple Inoculation

Unwaxed mature Granny Smith apples (medium size, ~220 g/apple) without cuts, bruising, or scars were selected and rinsed with cold tap water and dried overnight to balance apple temperature to room temperature (22 ± 1°C, RT). Apples were then inoculated with *L. monocytogenes* by submerging into the inoculum solution prepared above and gently agitating for 8 min to let bacteria evenly distribute on each apple as described previously (Sheng et al., 2017). Inoculated apples were stored at RT under environmental relative humidity for 24 or 48 h before being subjected to the PAA treatments. Meanwhile, apples were sampled right after inoculation, 24 and 48 h post-inoculation to confirm the established *L. monocytogenes* population.

### Antimicrobial Immersion Procedure

Bioside HS (EnviroTech, Modesto, CA, US) containing 15% of PAA was used to prepare solutions of 40, 60, and 80 ppm of PAA. All PAA solutions were prepared with tap water, unless otherwise specified. The concentration of PAA was verified using a titration kit (Aquaphoenix Scientific, Hanover, PA, US). Apples at 24 and 48 h post-inoculation were immersed in respective antimicrobial solutions with agitation for 30 s or 2 min; 10 apples were used per treatment. All treatments were repeated independently three times. PAA solutions were used at RT unless otherwise specified.

To evaluate the influence of pH on the efficacy of PAA, the pH of PAA solution was adjusted with 6.0 M HCl to achieve a pH of 2.5 and 3.8, and PAA dissolved in tap water had a pH of 6.3. Chlorine solution with 100 ppm free available chlorine (FAC) was used as a reference control and prepared from Accu-Tab (Pace International, Wapato, WA, US) (Beuchat and Ryu, 1997). The pH of a chlorine solution was adjusted to 6.8 with 6 M HCl before being used in apple treatment. Water wash of apples was used as negative control to show the bacterial reduction due to factors other than antimicrobial activities. The pH and the oxidation reduction potential (ORP) of solutions were measured with an Orion Versa Star Pro advanced electrochemistry meter (Thermo Scientific, Waltham, WA, US) with an 8302Bnumd Ross Ultra pH/ATC Triode and ORP Triode. FAC was confirmed with a Taylor K-2006 complete test kit (Taylor Technologies, Sparks, MD, US).

### Water Hardness Determination and Adjustment

Water hardness was measured by a hardness test kit (Hach, Loveland, CO, US). Three levels of water hardness (20, 140, and 460 ppm) were selected in this study to determine the influence of water hardness on PAA antimicrobial efficacy. Deionized water and tap water were used as water with 20 and 140 ppm hardness, respectively. Water with a hardness of 460 ppm was achieved by adding calcium chloride (Sigma, St Louis, MO, US) to tap water.

### Antimicrobial Efficacy of Peroxyacetic Acid at Elevated Temperature

To evaluate antimicrobial efficacies of PAA at elevated temperature, PAA solution was made with water preheated to the target temperatures (~22–49°C) and used immediately after preparation. The temperature of PAA solutions was maintained for each setting throughout the experiment. The concentration and the temperature of PAA were verified using a PAA titration kit (Aquaphoenix Scientific, Hanover, PA, US) and a thermometer (Fisher Scientific, Hampton, NH, US), respectively, before and after treatment. The temperature of the apple surface was measured with a digital thermometer with a probe (Fisher Scientific).

### Microbial Analysis of Apples

Immediately after antimicrobial treatment, each apple was individually placed into a sterile stomacher bag with 10-ml sterile PBS and hand-rubbed for 1.5 min to detach microbiota from apple surfaces. The detached microbial suspension was 10-fold serially diluted with sterile PBS, and 0.1 or 1 ml (333 μl/plate, 3 plates) from appropriate dilutions was plated on TSAYE plates overlaid with Modified Oxford agar (MOX, BD), and incubated at 35 ± 2°C for 48 h. Non-inoculated apples were processed the same way as inoculated apples and plated onto TSAYE for total plate count (TPC, BD) and Potato Dextrose Agar (PDA, BD) for yeasts and molds count (Y/M), respectively. TSAYE and PDA plates were incubated at 35 ± 2°C for 48 h and at RT for 5 days, respectively. The detection limit of all microorganisms was 10 CFU/apple.

### Statistical Analysis

Data were analyzed with one-way Analysis of Variance (ANOVA) using IBM SPSS 19.0 (Chicago, IL, US). Mean difference was discerned by Least Significant Difference (LSD) multiple comparison. *p* < 0.05 was considered statistically significant. Each experiment was repeated three times independently. For a selected independent test, there are 10 apples per treatment, where each apple is an experimental unit. Data were reported as mean ± SEM (standard error mean), *n* = 3.

## Results

### Influence of Concentration on the Antimicrobial Efficacy of Peroxyacetic Acid

**Figure 1:**
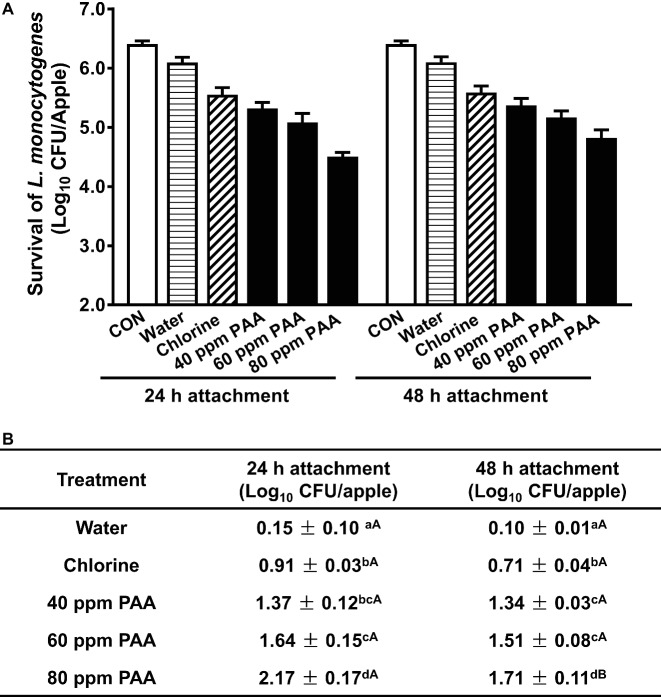
Antimicrobial efficacy of peroxyacetic acid (PAA) against *L. monocytogenes* on apples at a 2-min contact time at 22°C. **(A)** Representative bar graph of survival of *L. monocytogenes* on apples post-PAA treatment. **(B)** Log reduction of *L. monocytogenes* on apples, averaged from three independent experiments. ^a–d^Means within a column without common letter differ significantly (*p <* 0.05), ^A–B^means within a row without common letter differ significantly (*p <* 0.05). Mean ± SEM, *n* = 3. 24-h attachment: *L. monocytogenes* are allowed to attach to apples for 24 h before antimicrobial treatment; 48-h attachment: *L. monocytogenes* are allowed to attach to apples for 48 h before antimicrobial treatment.

Apples were inoculated with ~6.4 Log_10_ CFU/apple and then subjected to antimicrobial treatments after 24 and 48 h inoculation, respectively. At 24 h post-inoculation, 100 ppm chlorine at pH 6.8 caused 0.91 Log_10_ CFU/apple reduction and tap water wash led to 0.15 log reduction. PAA at 40 ppm reduced *L. monocytogenes* on fresh apples by 1.37 Log_10_ CFU/apple at 2-min exposure, which was more effective than that of 100 ppm chlorine (Figures 1A,B). Increasing PAA concentration significantly increased its bactericidal effects. PAA at 80 ppm and 2-min contact time reduced *L. monocytogenes* on apples by 2.17 Log_10_ CFU/apple (Figure 1B). Extending the postinoculation time from 24 to 48 h significantly reduced 80 ppm PAA efficacy with a log reduction of 1.71 Log_10_ CFU/apple at a 2-min treatment, though it had a minor influence on PAA efficacy at 40 and 60 ppm (Figure 1B). During the postharvest processing, foodborne pathogens can contaminate apples at any stage; thus, a bacterial attachment time of 48 h was used in the following study to mimic the harshest condition. PAA at 80 ppm was selected to mimic the current industry practice and to assess the maximal expected reduction.

### Impacts of Water Hardness and pH on Antimicrobial Efficacy of Peroxyacetic Acid

**Figure 2:**
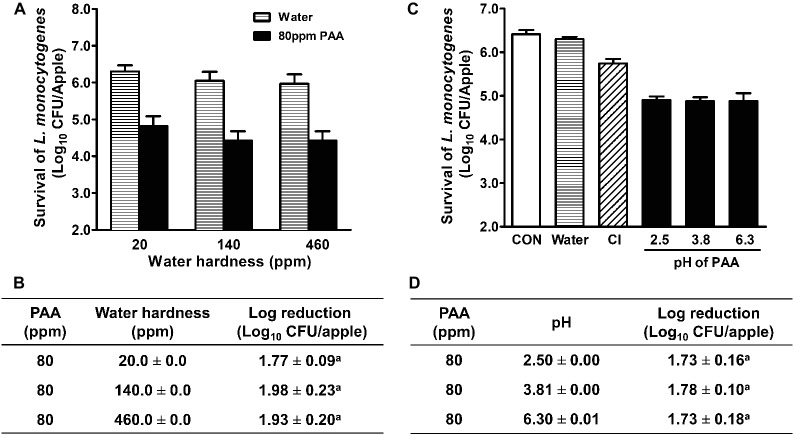
Antimicrobial efficacy of peroxyacetic acid (PAA) against *L. monocytogenes* on apples under different water hardness and pH at 22°C. *L. monocytogenes* are allowed to attach to apples for 48 h before antimicrobial treatment. **(A,C)** Representative bar graphs of *L. monocytogenes* survival on apples; **(B,D)** Log reduction of *L. monocytogenes* on apples, averaged from three independent experiments. ^a^Means within a column with common letter are not different significantly (*p <* 0.05), mean ± SEM, *n* = 3.

The hardness of wash water varies in the apple industry in Washington and ranges from 0 to 450 ppm (per our survey data). Thus, impacts of water hardness on PAA efficacy were further analyzed. PAA solutions made with water of different hardness had a similar efficacy against *L. monocytogenes* on fresh apples, ranging from 1.8 to 2.0 Log_10_ CFU/apple reduction (Figures 2A,B). Next, we examined the impact of pH on PAA antimicrobial efficacy and found that PAA exerted a similar bactericidal effect at pH 2.5–6.3, which reduced *L. monocytogenes* on apples by 1.7–1.8 Log_10_ CFU/apple (Figures 2C,D). In the subsequent studies, all PAA solutions were made with tap water with ~140 ppm hardness and a pH of 6.3.

### Improved Efficacy of Peroxyacetic Acid Against *L. monocytogenes* on Fresh Apples at Elevated Temperatures

**Figure 3:**
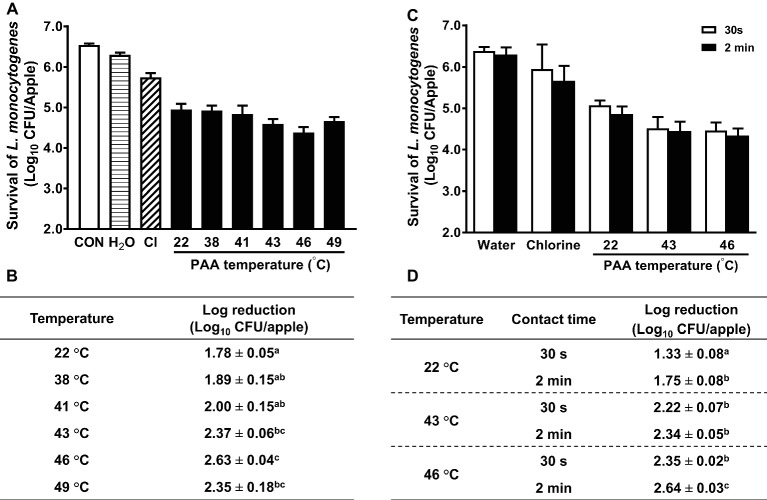
Influence of temperature and contact time on antimicrobial efficacy of peroxyacetic acid (PAA) against *L. monocytogenes* on apples. **(A,C)** Representative bar graphs of *L. monocytogenes* survival on apples. **(B,D)** Log reduction of *L. monocytogenes* on apples, averaged from three independent experiments. ^a–c^Means within a column or a temperature without common letter differ significantly (*p <* 0.05). Mean ± SEM, *n* = 3.

In some commercial apple packing operations, apples are subjected to a hot-water (up to 38°C) rinse before sanitizer intervention as a necessary treatment to facilitate application of waxes or fruit lusters. Studies also showed that a 40-min exposure to 50°C water had no negative effect on apple quality (Hansen et al., 2006). This prompted us to assess the antimicrobial efficacy of PAA against *L. monocytogenes* on apples at elevated temperatures. Increasing PAA solution temperature from RT to 41°C had no significant influence on antimicrobial efficacy of PAA (Figures 3A,B). However, when the temperature was further increased to 43°C, the reduction of *L. monocytogenes* was significantly improved (Figure 3B). PAA at 43 and 46°C reduced *L. monocytogenes* on apples by 2.37 ± 0.06 and 2.63 ± 0.04 Log_10_ CFU/apple, respectively (Figure 3B). However, increasing PAA solution temperature to 49°C failed to further enhance its effectiveness (Figure 3B). Reducing contact time from 2 min to 30 s decreased its bactericidal effects (Figures 3C,D). The concentration of PAA at all the tested temperatures remained stable during the wash treatment, while pH and ORP of PAA solutions gradually decreased with increased temperature (Table 1). The surface temperatures of apples post 2-min PAA treatment at 43 and 46°C were 37.4 ± 0.3 and 38.4 ± 0.4°C, respectively (Table 2).

**Table 1:** pH and oxygen reduction potential (ORP) of peroxyacetic acid (PAA) at different temperatures.

Temperature	PAA conc. (ppm)	pH	ORP (RmV)
22°C	80.0 ± 0.0	6.27 ± 0.01	375.0 ± 0.7
38°C	80.0 ± 0.0	6.24 ± 0.01	367.0 ± 0.9
41°C	80.0 ± 0.0	6.21 ± 0.01	363.2 ± 0.5
43°C	80.0 ± 0.0	6.20 ± 0.02	359.4 ± 0.5
46°C	80.0 ± 0.0	6.15 ± 0.03	359.4 ± 0.6
49°C	80.0 ± 0.0	6.02 ± 0.03	351.0 ± 0.6

*Data are presented as mean ± SEM, *n* = 3*.

**Table 2:** Temperature of apple surface and peroxyacetic acid (PAA) solution at pre- and post-PAA intervention.

Treatment	Apple surface T (°C)		PAA solution T (°C)	
	Before	After	Before	After
PAA (43°C, 30 s)	19.8 ± 0.0	34.8 ± 0.0	43.7 ± 0.2	42.8 ± 0.2
PAA (43°C, 2 min)	19.8 ± 0.0	37.4 ± 0.3	43.8 ± 0.2	42.5 ± 0.3
PAA (46°C, 30 s)	19.8 ± 0.0	36.3 ± 0.4	46.6 ± 0.1	45.3 ± 0.1
PAA (46°C, 2 min)	19.8 ± 0.0	38.4 ± 0.4	46.5 ± 0.0	45.3 ± 0.2

*Data are presented as mean ± SEM, *n* = 3*.

### Efficacy of Peroxyacetic Acid Against Background Microbiota at Elevated Temperatures

**Figure 4:**
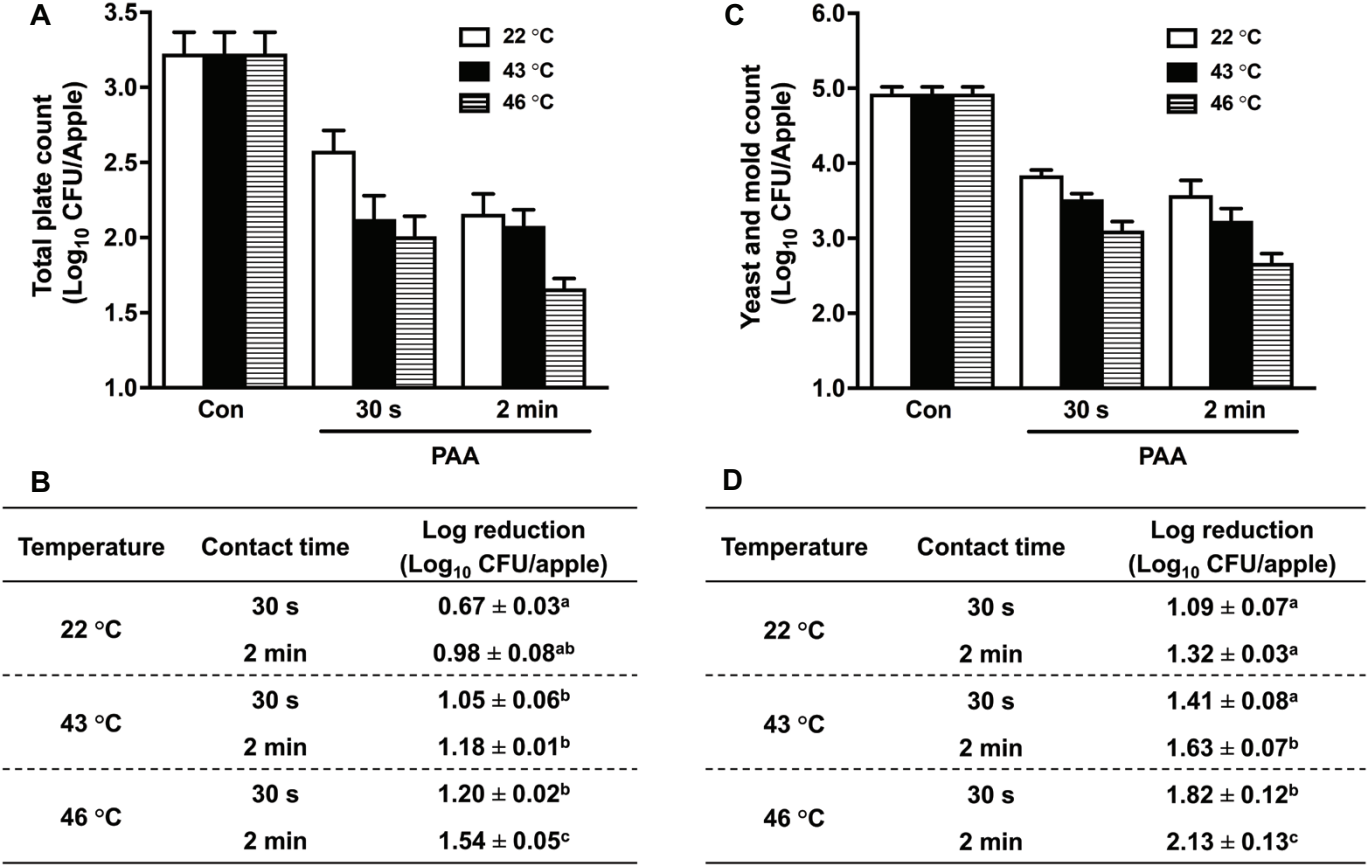
Efficacy of peroxyacetic acid (PAA) against background microbiota on apples treated at different temperatures. **(A)** Total plate count (TPC) of residential bacteria on apples. **(C)** Representative survival of yeast and mold (Y/M). **(B**,**D)** Log reduction of TPC **(B)** and YM **(D)** on apples, averaged from three independent experiments. ^a–c^Means within a column without common letter differ significantly (*p <* 0.05), mean ± SEM, *n* = 3.

We further evaluated the effectiveness of PAA in reducing apple resident microbiota. PAA at 46°C significantly improved its antimicrobial activity compared with that at RT, and reduced TPC by 1.20 ± 0.02 and 1.54 ± 0.05 Log_10_ CFU/apple at contact times of 30 s and 2 min, respectively (Figures 4A,B). Similarly, PAA at 46°C enhanced its effectiveness in reducing Y/M on apples and caused ~2.0 Log_10_ CFU/apple reduction at contact time of 2 min, which was almost double the log reduction when PAA was applied at RT (Figures 4C,D).

## Discussion

In the fresh produce industry, especially, the fresh apple packing industry, PAA has become the preferred antimicrobial for microbial interventions. PAA is currently allowed under the National Organic Program (NOP) for organic food handling (USDA, 2016). The advantages of using PAA over commonly used chlorine are the unnecessity to adjust pH, low reactivity with organic matter, and safety of its reaction and residual breakdown products (Kitis, 2004; Buchholz and Matthews, 2010).

### Antimicrobial Efficacy of Peroxyacetic Acid at Current Commercial Treatment Conditions

The efficacy of PAA against *L. monocytogenes* on apples is concentration dependent. Similarly, PAA at 25, 51, and 70 ppm for 3 min exposure resulted in 1.0, 1.4, and 1.8 Log_10_ CFU/g reduction of *L. monocytogenes* on bean sprouts, respectively (Neo et al., 2013). A PAA wash for 5 min at 80 and 250 ppm delivered a 0.4 and 1.3 Log_10_ CFU/g reduction of *L. monocytogenes* on iceberg lettuce, respectively (Baert et al., 2009). Under ambient temperature, 2-min wash with 80 ppm PAA delivered ~1.7 Log_10_ CFU/apple reduction of *L. monocytogenes* on apples, which was a little more effective than 80 ppm PAA against *L. monocytogenes* on Golden Delicious apples, where a log reduction time is about 80 s (Rodgers et al., 2004). *E. coli* O157:H7 on apples is less responsive to PAA, where 80 ppm PAA only reduced it by ~1.0 Log_10_ CFU/apple at 5-min contact time (Wisniewsky et al., 2000; Alcala et al., 2011). The difference in susceptibility could be due to difference in bacterial strains, surface attributes of apple varieties, as well as source of PAA solution.

The effectiveness of PAA against *L. monocytogenes* on apple surfaces was not measurably impacted by the hardness of water or pH condition. This is consistent with a previous publication that states that the stability of PAA solution was not affected by hardness of water (Artes et al., 2007). A 200 ppm PAA solution at both pH 2.8 and 4.3 reduced *Salmonella* Heidelberg on poultry product by ~1.0 Log_10_ CFU/ml at a 15-s contact time (Donabed, 2015). This might be due to the active compound of PAA solution, undissociated acid form of PAA, that was stable at pH equal to or less than its pKa of 8.2 (Yuan et al., 1997; Wagner et al., 2002), thus exhibiting a similar antimicrobial efficacy at the tested pH range. Antimicrobial action of PAA is possibly attributed to its action on the lipoproteins in the cell membrane, which results in disruption of the lipoprotein cytoplasmic membrane or cell walls due to oxidative stress, and subsequently denaturation of intracellular enzymes and other important macromolecules (Leaper, 1984; Maris, 1995).

Antimicrobial efficacy of 80 ppm PAA at an ambient temperature against *L. monocytogenes* on apples increased with increased contact time. There was ~0.42 more log reduction at a 2-min contact time compared to that of 30-s contact time. Similarly, PAA at 80 ppm for 5 min reduced native microorganisms on iceberg lettuce by ~2.4 Log_10_ CFU/g, which was ~0.9 Log_10_ CFU/g more reduction than that of a 2-min contact time (Vandekinderen et al., 2009). However, 70 ppm PAA at either 1.5- or 3-min contact time reduced *L. monocytogenes* on mung bean sprouts by ~1.8 Log_10_ CFU/g (Neo et al., 2013). 1- and 2-min PAA treatments at 75 ppm showed a comparable efficacy (~2 Log_10_ CFU/produce reduction) against *Salmonella* on bell peppers and cucumbers (Yuk et al., 2006).

### Enhanced Antimicrobial Efficacy of Peroxyacetic Acid at Elevated Temperature

The biocidal effects of PAA significantly increased when the PAA solutions were applied at 43–46**°**C compared with that at an ambient temperature. A similar phenomenon was observed on beef carcasses. Even at 1000 ppm, PAA showed a minimal efficacy against *E. coli* O157:H7 on beef carcasses when applied at RT, while it resulted in ~0.9 Log_10_ CFU/cm^2^ at when applied at 45°C (King et al., 2005). The elevated temperature might increase transportation of PAA across bacteria membranes, impairing intracellular osmotic balance, and subsequently facilitate cell death (Laroche et al., 2001; McCutcheon and Elimelech, 2006). Additionally, increasing wash solution temperature reduced the surface tension between hydrophobic apple surfaces and hydrophilic PAA solution, thus exposing the entrapped *L. monocytogenes* cells to PAA (Vandekinderen et al., 2009). PAA delivered stronger antimicrobial efficacy at non-dissociated form (Luukkonen et al., 2014), and PAA at 43–46°C was likely maintained the non-dissociated form and enhanced its antimicrobial efficacy against *L. monocytogenes.* Though the decomposition rate of PAA was negatively affected by increased temperature at long-time exposure (Kunigk et al., 2001), it had no influence on PAA concentration within minutes of exposure. The concentration of PAA maintained stable after 2-min intervention at respective temperatures.

Apple resident microbiota including TPC and Y/M are reported to affect apple fruit quality and shelf life during storage (Doores, 1983; Palou et al., 2009). At an ambient temperature, PAA at 80 ppm and 2-min contact time showed a limited antimicrobial efficacy (~1.0 log reduction) against TPC or Y/M. Similarly, 80 ppm PAA at 5 min reduced Y/M by 1.0–1.5 Log_10_ CFU/apple on apples when it was applied at an ambient temperature (Rodgers et al., 2004; Kreske et al., 2006). Similar to *L. monocytogenes*, elevation of PAA solution temperature significantly improved its biocidal effects against apple resident microbiota with more significant effect on Y/M lethality. Data indicate that PAA intervention at 43–46°C has a potential to increase apple shelf life in addition to improved microbial safety.

Elevated temperature slightly increased the surface temperature of apples to 35–38°C depending on treatment temperature and contact time. A previous study showed that 46°C treatment of apples for 12 h increased the firmness of fruits and reduced the development of superficial scald following subsequent 3 months under refrigerated storage at 0°C (Klein and Lurie, 1992). However, extended exposure time to 24 h resulted in fruit damage after storage (Klein and Lurie, 1992). Thus, temperate can have a negative effect for long-term exposure at evaluated temperature; however, PAA intervention at elevated temperatures used in this study was conducted in a short contact time, thus it has a minimal impact on apple fruit quality.

## Conclusion

PAA at 80 ppm and 2-min contact time reduced *L. monocytogenes* on fresh apples by ~1.7 Log_10_ CFU/apple when applied at an ambient temperature, which was not affected by the hardness or pH of PAA solution. PAA intervention at 43–46°C significantly enhanced its bactericidal effects, and reduced *L. monocytogenes* on fresh apples by 2.3–2.6 Log_10_ CFU/apple, and TPC and Y/M by ~1.5 and ~2.1 Log_10_ CFU/apple, respectively. These data provide valuable technical information and practical intervention methods for the apple packing and processing industry to support compliance with Food Safety Modernization Preventive Controls requirements. The study also provides important reference points for controlling other important foodborne pathogens such as *E. coli* O157:H7 and *Salmonella* on fresh apples, as well as other fresh produce with similar surface traits and postharvest handling systems.

## Author Contributions

XS, LS and HG performed the experiment. XS wrote the manuscript. IH, LS and TS revised the manuscript. IH provided the survey information. M-JZ guided the experimental design and revised the manuscript.

## Conflict of Interest Statement

The authors declare that the research was conducted in the absence of any commercial or financial relationships that could be construed as a potential conflict of interest.

## References

[ref1] Abadias M. Alegre I. Usall J. Torres R. Vinas I. (2011). Evaluation of alternative sanitizers to chlorine disinfection for reducing foodborne pathogens in fresh-cut apple. Postharvest Biol. Technol. 59, 289–297. 10.1016/j.postharvbio.2010.09.014

[ref2] Abadias M. Usall J. Oliveira M. Alegre I. Vinas I. (2008). Efficacy of neutral electrolyzed water (NEW) for reducing microbial contamination on minimally-processed vegetables. Int. J. Food Microbiol. 123, 151–158. 10.1016/j.ijfoodmicro.2007.12.008, PMID: 18237810

[ref3] Alcala P. E. Killinger K. Adhikari A. Mayer M. (2011). Effectiveness of Lactic Acid and Peroxyacetic Acid Treatments on Reducing Generic and Pathogenic E. coli on Fresh Apples. Available at: http://lfp.mme.wsu.edu/REU2012/files/30.pdf (Accessed August 1, 2018).

[ref4] Angelo K. M. Conrad A. R. Saupe A. Dragoo H. West N. Sorenson A.. (2017). Multistate outbreak of Listeria monocytogenes infections linked to whole apples used in commercially produced, prepackaged caramel apples: United States, 2014–2015. Epidemiol. Infect. 145, 848–856. 10.1017/S0950268816003083, PMID: 28065170 PMC6542465

[ref5] Artes F. Gomez P. Artes-Hernandez F. Aguayo E. Escalona V. (2007). Improved strategies for keeping overall quality of fresh-cut produce. Acta Hortic. 746, 245–258. 10.17660/ActaHortic.2007.746.27

[ref6] Baert L. Vandekinderen I. Devlieghere F. Van Coillie E. Debevere J. Uyttendaele M. (2009). Efficacy of sodium hypochlorite and peroxyacetic acid to reduce murine Norovirus 1, B40-8, Listeria monocytogenes, and *Escherichia coli* O157:H7 on shredded iceberg lettuce and in residual wash water. J. Food Prot. 72, 1047–1054. 10.4315/0362-028X-72.5.1047, PMID: 19517733

[ref7] Banach J. L. Sampers I. Van Haute S. van der Fels-Klerx H. J. (2015). Effect of disinfectants on preventing the cross-contamination of pathogens in fresh produce washing water. Inl. J. Environ. Res. Public Health 12, 8658–8677. 10.3390/ijerph120808658, PMID: 26213953 PMC4555240

[ref8] Beuchat L. R. Nail B. V. Adler B. B. Clavero M. R. (1998). Efficacy of spray application of chlorinated water in killing pathogenic bacteria on raw apples, tomatoes, and lettuce. J. Food Prot. 61, 1305–1311. 10.4315/0362-028X-61.10.1305, PMID: 9798146

[ref9] Beuchat L. R. Ryu J. H. (1997). Produce handling and processing practices. Emerg. Infect. Dis. 3, 459–465. 10.3201/eid0304.970407, PMID: 9366597 PMC2640071

[ref10] Brown D. Bridgeman J. West J. R. (2011). Predicting chlorine decay and THM formation in water supply systems. Rev. Environ. Sci. Biotechnol. 10, 79–99. 10.1007/s11157-011-9229-8

[ref11] Buchholz A. Matthews K. R. (2010). Reduction of Salmonella on alfalfa seeds using peroxyacetic acid and a commercial seed washer is as effective as treatment with 20000 ppm of Ca(OCl)_2_. Lett. Appl. Microbiol. 51, 462–468. 10.1111/j.1472-765X.2010.02929.x, PMID: 20840553

[ref12] Carrasco G. Urrestarazu M. (2010). Green chemistry in protected horticulture: the use of peroxyacetic acid as a sustainable strategy. Inl. J. Mol. Sci. 11, 1999–2009. 10.3390/ijms11051999, PMID: 20559497 PMC2885089

[ref13] Dell’Erba A. Falsanisi D. Liberti L. Notarnicola M. Santoroa D. (2007). Disinfection by-products formation during wastewater disinfection with peracetic acid. Desalination 215, 177–186. 10.1016/j.desal.2006.08.021

[ref14] Donabed J. (2015). The Efficacy of Peracetic Acid in Conjunction with Different Acid Blends Against Salmonella Heidelberg, Campylobacter Jejuni, Andaerobic Bacteria Inoculated Poultry. Available at: https://envirotech.com/wp-content/uploads/2017/04/PAA-Acid-report-FINAL.pdf (Accessed August 1, 2018).

[ref15] Doores S. (1983). The microbiology of apples and apple products. Crit. Rev. Food Sci. Nutr. 19, 133–149. 6380951 10.1080/10408398309527372

[ref16] FDA (2012). The Bad Bug Book: Foodborne Pathogenic Microorganisms and Natural Toxins Handbook. Available at: https://www.fda.gov/downloads/Food/FoodborneIllnessContaminants/UCM297627.pdf (Accessed August 1, 2018).

[ref17] FDA (2015). Northstar Produce Inc. Recalls Granny Smith Size 175 Apples Because of Possible Health Risk. Available at: https://fdarecall.wordpress.com/2015/10/30/northstar-produce-inc-recalls-granny-smith-size-175-apples-because-of-possible-health-risk/ (Accessed August 1, 2018).

[ref18] FDA (2016). Recalls, Market Withdrawals, & Safety Alerts - Fresh from Texas Recalls Apple Product Because of Possible Health Risk. Available at: https://www.fda.gov/Safety/Recalls/ucm494345.html (Accessed August 1, 2018).

[ref19] FDA (2017a). Code of Federal Regulations Title 21. Available at: https://www.accessdata.fda.gov/SCRIPTs/cdrh/cfdocs/cfcfr/CFRSearch.cfm?fr=173.315&SearchTerm=chemicals (Accessed August 1, 2018).

[ref20] FDA (2017b). Jack Brown Produce, Inc. Recalls Gala, Fuji, Honeycrisp and Golden Delicious Apples Due to Possible Health Risk. Available at: https://www.fda.gov/Safety/Recalls/ucm589722.html (Accessed August 1, 2018).

[ref21] Gehr R. Wagner M. Veerasubramanian P. Payment P. (2003). Disinfection efficiency of peracetic acid, UV and ozone after enhanced primary treatment of municipal wastewater. Water Res. 37, 4573–4586. 10.1016/S0043-1354(03)00394-4, PMID: 14568042

[ref22] Gil M. I. Marin A. Andujar S. Allende A. (2016). Should chlorate residues be of concern in fresh-cut salads? Food Control 60, 416–421. 10.1016/j.foodcont.2015.08.023

[ref23] Glass K. A. Golden M. C. Wanless B. J. Bedale W. Czuprynski C. (2015). Growth of Listeria monocytogenes within a caramel-coated apple microenvironment. MBio 6:e01232–15. 10.1128/mBio.01232-15, PMID: 26463161 PMC4620460

[ref24] Gonzalez R. J. Luo Y. Ruiz-Cruz S. McEvoy J. L. (2004). Efficacy of sanitizers to inactivate *Escherichia coli* O157:H7 on fresh-cut carrot shreds under simulated process water conditions. J. Food Prot. 67, 2375–2380. 10.4315/0362-028X-67.11.2375, PMID: 15553615

[ref25] Hansen J. D. Drake S. R. Heidt M. L. Watkins M. A. Tang J. Wang S. (2006). Radio frequency-hot water dips for postharvest codling moth control in apples. J. Food Process. Preserv. 30, 631–642. 10.1111/j.1745-4549.2006.00094.x

[ref26] Hellstrom S. Kervinen R. Lyly M. Ahvenainen-Rantala R. Korkeala H. (2006). Efficacy of disinfectants to reduce Listeria monocytogenes on precut iceberg lettuce. J. Food Prot. 69, 1565–1570. 10.4315/0362-028X-69.7.1565, PMID: 16865887

[ref27] Hua M. Y. Chen H. C. Tsai R. Y. Lin Y. C. (2011). A novel amperometric sensor for peracetic acid based on a polybenzimidazole-modified gold electrode. Electrochim. Acta 56, 4618–4623. 10.1016/j.electacta.2011.02.092

[ref28] King D. A. Lucia L. M. Castillo A. Acuff G. R. Harris K. B. Savell J. W. (2005). Evaluation of peroxyacetic acid as a post-chilling intervention for control of *Escherichia coli* O157: H7 and Salmonella Typhimurium on beef carcass surfaces. Meat Sci. 69, 401–407. 10.1016/j.meatsci.2004.08.010, PMID: 22062977

[ref29] Kitis M. (2004). Disinfection of wastewater with peracetic acid: a review. Environ. Int. 30, 47–55. 10.1016/S0160-4120(03)00147-8 14664864

[ref30] Klein J. D. Lurie S. (1992). Prestorage heating of apple fruit for enhanced postharvest quality: interaction of time and temperature. HortScience 27, 326–328. 10.21273/HORTSCI.27.4.326

[ref31] Kreske A. C. Ryu J. H. Beuchat L. R. (2006). Evaluation of chlorine, chlorine dioxide, and a peroxyacetic acid-based sanitizer for effectiveness in killing Bacillus cereus and *Bacillus thuringiensis* spores in suspensions, on the surface of stainless steel, and on apples. J. Food Prot. 69, 1892–1903. 10.4315/0362-028X-69.8.1892, PMID: 16924915

[ref32] Kunigk L. Gomes D. R. Forte F. Vidal K. P. Gomes L. F. Sousa P. F. (2001). The influence of temperature on the decomposition kinetics of peracetic acid in solutions. Braz. J. Chem. Eng. 18, 217–220. 10.1590/S0104-66322001000200009

[ref33] Laroche C. Beney L. Marechal P. A. Gervais P. (2001). The effect of osmotic pressure on the membrane fluidity of *Saccharomyces cerevisiae* at different physiological temperatures. Appl. Microbiol. Biotechnol. 56, 249–254. 10.1007/s002530000583, PMID: 11499939

[ref34] Leaper S. (1984). Influence of temperature on the synergistic sporicidal effect of peracetic acid plus hydrogen peroxide on *Bacillus subtilis* SA22 (NCA 72–52). Food Microbiol. 1, 199–203. 10.1016/0740-0020(84)90034-0

[ref35] Luukkonen T. Teeriniemi J. Prokkola H. Ramo J. Lassi U. (2014). Chemical aspects of peracetic acid based wastewater disinfection. Water SA 40, 73–80. 10.4314/wsa.v40i1.9

[ref36] Maris P. (1995). Modes of action of disinfectants. Rev. Sci. Tech. 14, 47–55. 10.20506/rst.14.1.829, PMID: 7548971

[ref37] McCutcheon J. R. Elimelech M. (2006). Influence of concentrative and dilutive internal concentration polarization on flux behavior in forward osmosis. J. Membr. Sci. 284, 237–247. 10.1016/j.memsci.2006.07.049

[ref38] Monarca S. Feretti D. Zerbini I. Zani C. Alberti A. Richardson S. D. (2002a). Studies on mutagenicity and disinfection by-products in river drinking water disinfected with peracetic acid or sodium hypochlorite. Water Sci. Tech.-W. Sup. 2, 199–204. 10.2166/ws.2002.0103 11833799

[ref39] Monarca S. Richardson S. D. Feretti D. Grottolo M. Thruston A. D. Zani C. (2002b). Mutagenicity and disinfection by-products in surface drinking water disinfected with peracetic acid. Environ. Toxicol. Chem. 21, 309–318. 11833799

[ref40] Neo S. Y. Lim P. Y. Phua L. K. Khoo G. H. Kim S. J. Lee S. C.. (2013). Efficacy of chlorine and peroxyacetic acid on reduction of natural microflora, *Escherichia coli* O157:H7, *Listeria monocytogenes* and Salmonella spp. on mung bean sprouts. Food Microbiol. 36, 475–480. 10.1016/j.fm.2013.05.001, PMID: 24010631

[ref41] Palou L. Smilanick J. L. Crisosto C. H. (2009). Evaluation of food additives as alternative or complementary chemicals to conventional fungicides for the control of major postharvest diseases of stone fruit. J. Food Prot. 72, 1037–1046. 10.4315/0362-028X-72.5.1037, PMID: 19517732

[ref42] Rodgers S. L. Cash J. N. Siddiq M. Ryser E. T. (2004). A comparison of different chemical sanitizers for inactivating *Escherichia coli* O157:H7 and *Listeria monocytogenes* in solution and on apples, lettuce, strawberries, and cantaloupe. J. Food Prot. 67, 721–731. 10.4315/0362-028X-67.4.721, PMID: 15083724

[ref43] Sheng L. Edwards K. Tsai H. C. Hanrahan I. Zhu M. J. (2017). Fate of Listeria monocytogenes on fresh apples under different storage temperatures. Front. Microbiol. 8:1369. 10.3389/fmicb.2017.01396, PMID: 28790993 PMC5522875

[ref44] USAA (2017). Us Apple Reproduction and Consumption Facts and Figures. Available at: http://usapple.org/wp-content/uploads/2016/02/USAppleToolKit-Production-Consumption.pdf (Accessed August 1, 2018).

[ref45] USAA (2018). Annual US Apple Crop Stats. Available at: http://usapple.org/all-about-apples/apple-industry-statistics/ (Accessed August 1, 2018).

[ref46] USDA (2016). Peracetic Acid Handling and Processing. Available at: https://www.ams.usda.gov/sites/default/files/media/Peracetic%20Acid%20TR%203_3_2016%20Handling%20final.pdf (Accessed August 1, 2018).

[ref47] Vandekinderen I. Devlieghere F. De Meulenaer B. Ragaert P. Van Camp J. (2009). Optimization and evaluation of a decontamination step with peroxyacetic acid for fresh-cut produce. Food Microbiol. 26, 882–888. 10.1016/j.fm.2009.06.004, PMID: 19835776

[ref48] Wagner M. Brumelis D. Gehr R. (2002). Disinfection of wastewater by hydrogen peroxide or peracetic acid: development of procedures for measurement of residual disinfectant and application to a physicochemically treated municipal effluent. Water Environ. Res. 74, 33–50. 10.2175/106143002X139730, PMID: 11995865

[ref49] Wang H. Feng H. Luo Y. G. (2006). Dual-phasic inactivation of *Escherichia coli* O157: H7 with peroxyacetic acid, acidic electrolyzed water and chlorine on cantaloupes and fresh-cut apples. J. Food Saf. 26, 335–347. 10.1111/j.1745-4565.2006.00053.x

[ref50] Wisniewsky M. A. Glatz B. A. Gleason M. L. Reitmeier C. A. (2000). Reduction of *Escherichia coli* O157: H7 counts on whole fresh apples by treatment with sanitizers. J. Food Prot. 63, 703–708. 10.4315/0362-028X-63.6.703, PMID: 10852561

[ref51] Yuan Z. Ni Y. Van Heiningen A. (1997). Kinetics of the peracetic acid decomposition: part II: pH effect and alkaline hydrolysis. Can. J. Chem. Eng. 75, 42–47. 10.1002/cjce.5450750109

[ref52] Yuk H. G. Bartz J. A. Schneider K. R. (2006). The effectiveness of sanitizer treatments in inactivation of Salmonella spp. from bell pepper, cucumber, and strawberry. J. Food Sci. 71, M95–M99. 10.1111/j.1365-2621.2006.tb15638.x

